# Innovative Application of Electroslag Remelting in Inclusion Removal from Silicon Alloys and Silicon Recovery from Waste Photovoltaic Modules

**DOI:** 10.3390/ma19102002

**Published:** 2026-05-12

**Authors:** Xianhui Wu, Hongbing Peng, Jie Zhou, Sheng Pang, Minghui He, Ruili Zheng, Houyuan Zhang, Dong Wang, Guoyu Qian, Zhi Wang

**Affiliations:** 1 School of Metallurgy Engineering, Jiangsu University of Science and Technology, Zhangjiagang 215600, China; 2 CAS Key Laboratory of Green Process and Engineering, National Engineering Research Center for Green Recycling of Strategic Metal Resources, Institute of Process Engineering, Chinese Academy of Sciences, Beijing 100190, China; 3 School of Chemical Engineering, University of Chinese Academy of Sciences, Beijing 100049, China

**Keywords:** photovoltaic module recycling, pyrolysis, electroslag remelting, separation and purification, inclusion removal

## Abstract

The rapid expansion of crystalline silicon photovoltaic (PV) modules has increased the demand for sustainable and high-value recycling strategies for end-of-life (EOL) modules. A significant challenge is the removal of impurities such as carbon, oxygen, and non-metallic inclusions introduced into silicon solar cells during the dissociation of PV laminates. To address this, we propose a non-consumable electrode electroslag remelting (NCE-ESR) process to effectively eliminate inclusions. In this process, the reverse flow of alloy droplets and the extensive contact area are crucial during the reverse flow slag washing. Initially, we studied the occurrence characteristics of inclusions in silicon solar cells obtained after pyrolysis from enterprises. Pyrolysis facilitated the formation of inclusions like Si-O, C-O, Al-O, and Si-N, particularly in the fine size range below 5 μm. To enhance impurity removal, the recycled Si was alloyed with Cu, which increased the melt density and impurity activity. Based on optimized thermodynamics and physical properties, we designed a novel electroslag composition of 40%CaO-40%SiO_2_-20%CaF_2_ suitable for silicon alloy refining. Notably, during the reverse flow slag washing of the Cu-Si alloy, the maximum removal rate of inclusions reached 77.42%. The average diameter of inclusions was reduced to below 6 μm, and the removal rates of impurity elements such as Al, O, and C exceeded 98.09%, 94.86%, and 86.08%, respectively. Finally, we independently developed the NCE-ESR equipment and conducted a kilogram-scale amplification test. The results indicated that the impurity removal rates of Al and O exceeded 97%, and the final inclusion size was less than 10 μm. This study demonstrates a scalable and environmentally friendly approach for the high-value recycling of silicon resources from decommissioned PV modules.

## 1. Introduction

As fossil fuel resources dwindle, solar energy emerges as a crucial, sustainable, and environmentally friendly alternative [1]. The solar photovoltaic (PV) industry has seen remarkable growth, with global installed PV capacity reaching 760.4 GW by 2020 and continuing to increase annually [2]. However, this expansion brings challenges in waste management [3,4]. A 2023 survey projected that China would utilize approximately 0.35 gigawatts of disassembled and scrapped components, equating to about 20,400 tons. The mass of decommissioned components is expected to reach 63,800 tons [5], presenting a significant challenge. Thus, managing decommissioned PV modules effectively has become a critical issue for the industry [6]. The rapid growth in PV capacity underscores the urgent need to address the disposal of end-of-life (EoL) PV modules, a central concern for the industry [5,7,8].

Improper landfilling of PV module waste is unacceptable today due to the leaching of harmful heavy metals [9,10]. In contrast, the collection and recycling of PV modules are becoming increasingly essential. Reusing high-value silicon wafers and recycling metals from EoL PV modules are vital for reducing environmental risks, lowering production costs, and fostering the sustainable growth of the PV industry [11,12,13,14]. Crystalline silicon (c-Si) PV modules dominate the global market, accounting for over 95% of all PV module types [15,16]. These modules typically use ethylene-vinyl acetate copolymer (EVA) as a sealant, which bonds the glass and solar cells during a high-temperature lamination process [17]. To disassemble c-Si PV modules, it is necessary to either completely remove the encapsulant or compromise its adhesive properties [18,19,20].

Current separation methods for c-Si PV modules are classified into mechanical, pyrolytic, and chemical techniques [21,22,23]. Mechanical methods encompass conventional crushing [24,25,26], high-pressure pulse crushing [23,27], and hot knife cutting. Crushing initiates component separation, while basic sorting produces fine particulate matter. The hot-knife technique uses a heated blade to melt the EVA layer, facilitating the separation of glass from other PV module materials. However, these methods often fail to completely separate layers, leaving EVA residues on the cells [28]. This issue reduces both material recovery rates and purity. Chemical processing methods are mainly divided into inorganic acid dissolution and organic reagent swelling or dissolution [29]. Organic solvents, in particular, merit attention. Various solvents, such as toluene, o-dichlorobenzene, and trichloroethylene, have been used to dissolve or swell EVA films. Research shows these solvents are absorbed by EVA, expanding and weakening adhesive bonds, which leads to interlayer separation [30,31]. Nevertheless, due to their high saturated vapor pressure, this separation process is limited to room temperature and can take several days [32]. Furthermore, organic solvents are highly toxic and generate significant mixed organic wastewater during EVA dissolution. Pyrolysis involves heating PV modules in different atmospheres to efficiently remove the organic encapsulant EVA, achieving interlayer separation [13,33]. This method effectively decomposes high-adhesion EVA films, with a decomposition rate exceeding 99% [34,35], making it the most direct and efficient approach for separating PV module layers. However, crystalline silicon PV modules often contain fluorine-based back sheets. Direct pyrolysis of these modules releases harmful substances like fluorides and dioxins, which damage the environment and equipment [33,36]. Typically conducted in air, pyrolysis can cause residual adhesive film substances to accumulate on silicon solar cells, increasing carbon-oxygen impurities, leading to more inclusions, and complicating silicon purification. Despite its efficiency, pyrolysis poses challenges with inclusions in silicon cells, necessitating immediate attention. Therefore, developing an environmentally friendly and efficient impurity removal method is crucial for achieving high-purity silicon recovery.

Several methods have been employed to remove inclusions [37]. Wang et al. utilized the combined effects of force and heat from high-frequency electromagnetic separation to efficiently eliminate 1–2 μm non-metallic inclusions from steel [38]. High-gravity separation has also effectively filtered out spinel and coarse Al_3_Ti inclusions from molten aluminum [39]. However, these methods are energy-intensive and impractical for industrial-scale applications. In the slag refining (SLR) process, integrating slag phase filtration with slag adsorption effectively removes inclusions, resulting in high-quality metals. Liu et al. developed a quinary slag system composed of Al_2_O_3_, CaO, SiO_2_, MgO, and CaF_2_ to enhance inclusion removal and improve the desulfurization process for 316L stainless steel [40]. While SLR effectively purifies metals, it does not regulate the microstructure of alloys. An ideal alloy purification method should regulate microstructure, conserve energy, be easy to operate, and achieve a high impurity removal yield [41].

Electroslag remelting (ESR) effectively purifies alloys and enhances their strength through slag refining and sequential solidification processes [42,43]. Previous research has shown that ESR meets the thermodynamic and kinetic conditions necessary for slag refining, effectively eliminating inclusions in silicon. However, challenges arise in designing the electroslag system for silicon purification. Typically, the electroslag used in steel-making belongs to the CaO-Al_2_O_3_-CaF_2_ series, known for its excellent electrical conductivity, density, and viscosity [44,45,46]. In contrast, silicon refining slag generally follows a CaO-SiO_2_-X system, characterized by a relatively high SiO_2_ content [46,47]. Evaluating whether this system meets the performance requirements of electroslag is crucial. Additionally, the viscosity of silicon closely matches that of the slag (2.3–2.6 g/cm^3^) [48], causing Si droplets to become encapsulated within the slag, hindering their passage through the slag layer. This study proposes alloying silicon with copper to enhance the density of silicon alloys. Incorporating copper not only increases the activity of impurities in silicon, facilitating their removal, but also reduces the likelihood of reactions with the slag. The concept of using copper alloying as an intermediate step in silicon purification is not entirely new. Mitrašinović et al. [49] introduced a method for purifying metallurgical-grade silicon to solar-grade silicon through copper alloying, effectively removing impurities and reducing multiple element contents in silicon to below the ICP detection limit, offering a cost-effective approach for solar-grade silicon preparation. Subsequently, Huang et al. [50] explored the formation of the Si-Cu alloy phase by alloying low-purity MG-Si with copper and treating the Si-Cu alloy with CaO-SiO_2_-CaCl_2_ slag to remove impurities, achieving further removal of B and P. Moreover, silicon alloy’s inherent brittleness complicates its use as a consumable electrode. This study innovates by employing a non-consumable graphite electrode (NCE), allowing the silicon alloy to traverse the slag layer as droplets or lumps, ensuring their passage through the slag layer.

From a sustainability standpoint, the ESR strategy discussed in this paper offers both potential benefits and limitations. ESR facilitates inclusion removal and melt purification through slag-metal interaction at high temperatures, and prior research has shown its effectiveness in purifying and recycling silicon waste. However, as a high-temperature electrothermal process, ESR involves significant energy consumption, which cannot be overlooked. Compared to recycling methods heavily dependent on acid leaching or solvent treatment, this paper suggests that ESR may have a sustainability edge by minimizing the use of chemical reagents and reducing liquid waste. However, it does not claim ESR as the environmentally superior option without quantitative evidence. The overall environmental benefits of ESR require further validation through energy consumption analysis or life cycle assessment.

This study investigates the effectiveness of Cu-Si alloys, derived from silicon solar cells through the pyrolysis of EoL PV modules and combined with copper, in eliminating non-metallic inclusions. A novel ternary electroslag system was designed to purify the Cu-Si alloy using theoretically calculated electroslag parameters. A reverse flow slag washing experiment was conducted on the Cu-Si alloy, which is central to ESR. In this process, Cu-Si alloy droplets descend from the slag pool and travel through the slag layer, while the slag moves upward relative to the droplets, creating a reverse flow slag washing scenario. The study identified optimal conditions for removing inclusions through reverse flow slag washing by examining how factors such as alloy particle size and composition affect inclusion removal efficiency. The feasibility of removing inclusions from Cu-Si alloy via ESR was confirmed through scale-up experiments. This research establishes a theoretical basis for the high-yield ESR of low-conductivity Cu-Si alloys, offering an efficient and eco-friendly approach for the collaborative, high-value recycling of silicon resources from decommissioned PV modules.

## 2. Experimental

### 2.1. Pyrolytic Delamination of Crystalline Silicon PV Cells

The EoL double-glass c-Si PV modules analyzed in this study originated from Zhejiang Jinko Energy Co., Ltd. (Haining, China), specifically model JKM535M-72HL4-V. Initially, the aluminum frames and junction boxes of the PV modules were manually removed to obtain the retired PV laminates required for the experiment. A hand-held cutter (MNT-070309, Shanghai Meinaite Industry Co., Ltd., Shanghai, China) was used to cut the laminates into fragments approximately 40 × 40 mm in size, ensuring uniform mass distribution. During this cutting process, the tempered glass and c-Si cells at the edges were inevitably damaged. To explore the effects of various pyrolysis conditions on the oxidation and burn-off of valuable metals in c-Si cells, as well as the characteristics of inclusions, the laminate fragments were placed in a graphite crucible and inserted into a tube furnace (GSL-1700X, Hefei Kejing Materials Technology Co., Ltd., Hefei, China). Pyrolysis was conducted at temperatures of 450 °C, 550 °C, and 650 °C under air, argon, and vacuum conditions, respectively, resulting in the separation of the pyrolyzed c-Si cells. The separated cells were then immersed in toluene in a water bath (SHJ-4, Suzhou Guofei Laboratory Co., Ltd., Suzhou, China) at 90 °C for 4 h. Subsequently, the c-Si cells were manually peeled off from the adhesive film. To remove any residual toluene from the cell surfaces, they were washed with ethanol and deionized water, followed by drying in a vacuum oven (DZF-6050, Shanghai Yiheng Scientific Instrument Co., Ltd., Shanghai, China) for 8 h. This served as a control experiment prior to pyrolysis.

Silicon solar cells, both before and after pyrolysis, underwent treatment with HNO_3_ and HF to measure the concentrations of Ag, Al, Si, Ti, and Sn. This analysis was conducted using an inductively coupled plasma optical emission spectrometer (ICP-OES, Optima 5300DV, PerkinElmer, Shelton, CT, USA). For the determination of elements such as C and O, an oxygen-nitrogen-hydrogen analyzer (ONH-2000, ELTRA GmbH, Haan, Germany) and a carbon-sulfur analyzer (CS-800, ELTRA GmbH, Haan, Germany) were used. Additionally, a field-emission scanning electron microscope (SEM, JSM-7800, JEOL Ltd., Tokyo, Japan) equipped with EDS (OXFORD IET 200) was employed. Sixty regions were randomly selected at a fixed magnification of 1000× to observe and statistically analyze the types, quantities, and size distributions of inclusions in c-Si solar cells, both pre- and post-pyrolysis.

### 2.2. Reverse Flow Slag Washing Refining Experiment

This study’s experimental component involved a two-stage slag refining process to remove impurities. Static slag refining tests used metallic Si and Cu-Si alloy as raw materials. By analyzing the oxidation states of Si and the alloy after static slag refining, we determined the optimal electroslag system and experimental conditions for reverse flow slag washing refining. Traditional silicon-based molten slag refining primarily relies on the CaO-SiO_2_ system. This study uses the CaO-SiO_2_ system as a foundation to identify a suitable slag system for countercurrent slag washing refining of silicon-based alloys. CaF_2_ is selected as a flux to lower the system’s melting point and viscosity. The CaO-SiO_2_-CaF_2_ slag system is employed to study slag refining for inclusion removal. Research indicates that when the molten slag’s basicity is 1, impurity removal efficiency in silicon is relatively high. Consequently, the slag system is designed with a basicity of 1 as a benchmark, with basicities of 0.5 and 2 serving as control groups for comparative analysis. To ensure the refining slag’s melting point remains below the alloy refining temperature of 1550 °C, the slag composition must be controlled. Marie-Aline et al.’s research on the CaO-SiO_2_-CaF_2_ slag system shows that if CaF_2_ content does not exceed 40 wt% [51], the slag system’s melting point stays below 1550 °C, meeting refining slag requirements. Considering both molten slag basicity and melting point, the initial composition of the CaO-SiO_2_-CaF_2_ slag system is designed, as detailed in Table 1.

**Figure 1:**
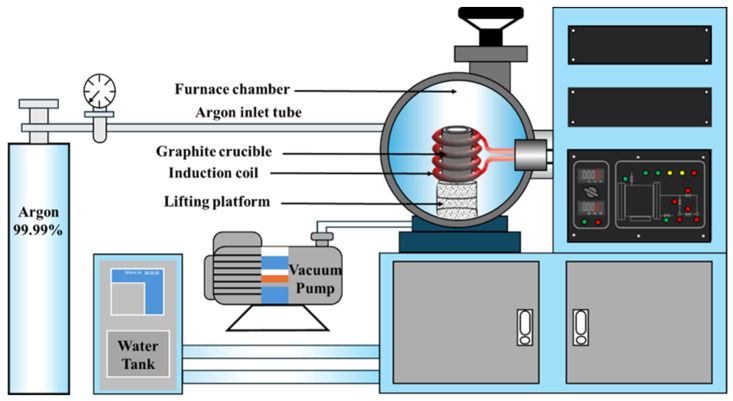
Schematic of the MZG vacuum induction melting furnace [41].

Individual pieces of industrial silicon (Si) weighing 1, 3, and 5 g were placed in graphite crucibles along with five types of pre-melted slags, each weighing 30 g. These samples were heated in an argon-protected atmosphere using an MZG series vacuum high-frequency induction furnace (IMCS-2100, Hefei Kejing Materials Technology Co., Ltd., Hefei, China). The temperature was steadily increased to 1550 °C and maintained for 20, 40, and 60 min before cooling. The optimal slag system, which minimized Si oxidation, was then used to further investigate the regulatory effect of the slag-alloy interface on alloy burning loss. Cu-Si alloy pieces weighing 3, 5, and 8 g were smelted with slag agents weighing twice as much as the alloys. These were also held at 1550 °C ± 20 °C for 20 min in an argon environment before cooling. To examine the impact of alloy size on inclusion removal, Cu-Si alloys with equivalent diameters of 0.579, 0.686, and 0.803 cm and a copper content of 50% were smelted with the optimal slag system at a slag-alloy mass ratio of 2:1 under the same conditions. Finally, under the optimal equivalent diameter, slag-alloy mass ratios were adjusted to 1:1, 2:1, 3:1, 4:1, and 5:1. After holding at 1550 °C for 20 min, the samples were cooled to room temperature, as illustrated in Figure 1.

**Figure 2:**
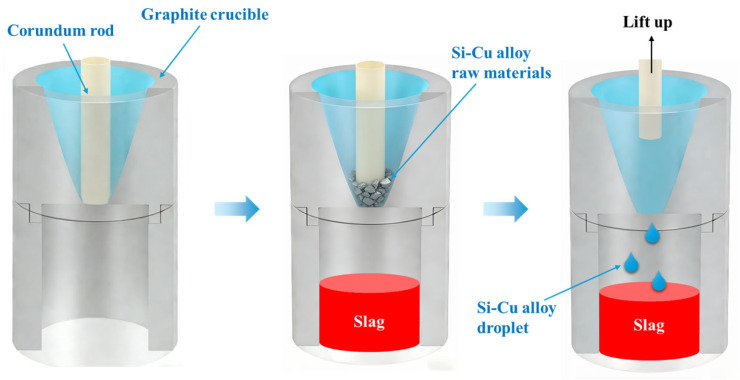
Composite graphite crucible structure schematic diagram.

A composite graphite crucible was designed for reverse flow slag washing and refining experiments of Cu-Si alloy, as shown in Figure 2. The crucible consists of an upper and a lower section, which are connected and secured by threads. The upper crucible has a central circular hole with a diameter of 10 mm. To ensure droplets enter this hole smoothly and drip into the lower crucible without obstruction, the upper crucible is inclined at an angle greater than 53°, and the hole channel height is 10 mm. To examine the effects of the alloy’s equivalent diameter and Cu content on inclusion removal, we utilized the optimal slag system composition and holding time identified in previous slag refining experiments. Cu-Si alloys with equivalent diameters of 0.579 cm, 0.686 cm, and 0.803 cm, and Cu contents of 30%, 50%, and 70%, were selected based on the optimal slag-to-alloy mass ratio and placed in the composite graphite crucible. The prepared Cu-Si alloy was loaded into the upper crucible, and the central hole was blocked with a solid corundum rod measuring 12 mm in diameter. The slag agent was placed in the lower crucible, and the upper and lower sections were tightened to secure the connection. This assembled crucible was then placed in a vacuum induction furnace and heated to 1550 °C. After a 20-min reaction, it was cooled to room temperature. Finally, the composite graphite crucible was removed, and the Cu-Si alloy samples, post reverse flow slag washing and refining, were separated.

The refined Cu-Si alloy samples underwent surface polishing with a handheld grinder to remove impurities. The alloy’s mass was determined both before and after refining using an electronic balance. Samples were bisected with an automatic cutter, and one half was chosen for testing. For electron microscope observation, samples from the Cu-Si alloys were prepared in three states: pre-refined, post-slag refining, and post-reverse flow slag washing refining. These surfaces were ground and polished, with magnification set at 1000×. Sixty identical fields were randomly selected for SEM and EDS tests to evaluate the number, size, and types of inclusions in the alloy. Furthermore, 1 g of the alloy from each refining stage—before refining, after slag refining, and after reverse flow slag washing refining—was immersed in an aqua regia solution, consisting of H_2_SO_4_, HF, and HNO_3_ in a specific ratio, for digestion. The elemental contents of Cu, Si, and Al were analyzed using ICP-OES, while C and O contents were measured using an oxygen-nitrogen-hydrogen analyzer and a carbon-sulfur analyzer.

### 2.3. System Scale-Up Experiment of NCE-ESR Refining

Given the physical properties of Cu-Si alloys, such as their high melting point, density, brittleness, and resistance to oxidation, we calculated various physical parameters of the electroslag system. These calculations focused on slag systems specifically designed for slag refining and reverse flow slag washing refining, ensuring they met the physical performance requirements essential for the ESR refining process. In this study, we determined the density of the slag system using the empirical formula proposed by Kazumi Ogino et al. [52], as shown in Equation (1). We also estimated the density of Cu-Si alloys with varying Cu contents at 1550 °C using the reaction module of Factsage. The electrical conductivity of the slag system was determined using the empirical formula modified by Dong Yanwu and Jiang Zhouhua et al. [53], as shown in Equation (2). Finally, we calculated the viscosities of the slag system at various temperatures using the Factsage software (Factsage 8.1).

100/ρ = 0.415(%CaF_2_) + 0.286(%CaO) + 0.367(%MgO) + 0.533(%SiO_2_) + 0.426(%TiO_2_) + 0.370(%ZrO_2_) + 0.742(%Na_2_O) + 0.530(%NaF) + 0.417(%Al_2_O_3_) * or 0.329(%Al_2_O_3_) ** (1)

(* CaO-Al_2_O_3_ based slags, ** CaF_2_-based slags).

K = 100 exp(1.911 − 1.38X_x_ − 5.69X^2^_x_) + 0.39(T − 1973) (2)

where X_x_ = x(Al_2_O_3_) + 0.2x(CaO) + 0.8x(MgO) + 0.75x(SiO_2_) + 0.5(x(TiO_2_) + x(ZrO_2_)); x(Al_2_O_3_) = 0–0.5; x(CaO) = 0–0.65; x(MgO) = 0–0.1; x(SiO_2_) = 0–0.17; x(TiO_2_) = 0–0.18; x(ZrO_2_) = 0–0.15. K—Melt pool conductivity, Ω^−1^⋅cm^−1^; T—Temperature, K.

Commercial-scale experiments were conducted in ambient air using an ESR furnace equipped with a graphite non-consumable electrode. The arc-striking material was centrally placed at the bottom of the mold, with 20 g of powdered electroslag evenly distributed around it. The electrode rod was securely positioned in the electroslag furnace’s electrode slot. Due to the high brittleness of the Cu-Si alloy, preparing it as a consumable electrode was challenging. To address this issue, we developed a novel ESR refining method employing a non-consumable electrode. Specifically, a highly conductive graphite electrode was used for current conduction, while Cu-Si alloy particles were directly added to the mold for melting. We utilized a total of 100 g of Cu-Si alloy particles, each with an equivalent diameter of 0.579 cm, as the experimental raw material.

After the experiment, we collected the refined Cu-Si alloy samples. These samples were leached using an aqua regia solution composed of H_2_SO_4_, HNO_3_, and HF in specific proportions. We measured the concentrations of elements such as Cu, Si, and Al in the extracted samples using ICP-OES. Additionally, we analyzed the levels of O and C in the alloy with an oxygen-nitrogen-hydrogen analyzer and a carbon-sulfur analyzer.

## 3. Results and Discussions

### 3.1. Metal Burning Behavior and Inclusion Characteristics During the Pyrolysis Stage

This section systematically examines the impact of the pyrolysis stage on the burning loss behavior of valuable metals in silicon solar cells and the state of inclusions. We investigate changes in metal content and the morphological evolution of inclusions under different pyrolysis atmospheric conditions. The analysis highlights the degree of burning loss of key valuable metals during pyrolysis, along with the quantity, type, and distribution characteristics of the inclusions.

**Figure 3:**
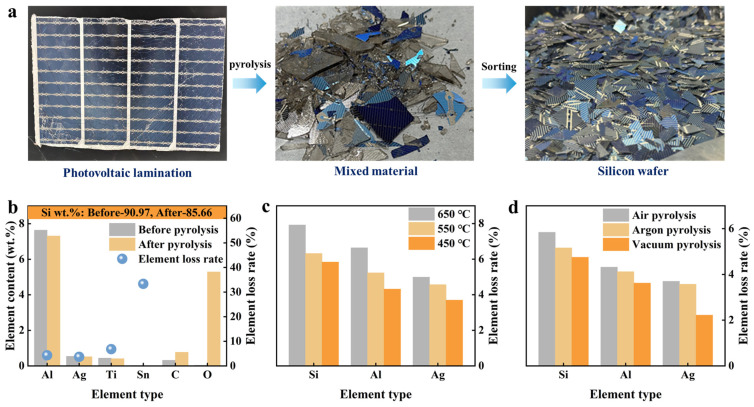
The influence of pyrolysis on the morphology and element loss of photovoltaic modules and solar cells: (**a**) Morphologies of components and solar cells before and after pyrolysis, (**b**) alterations in the concentrations of primary metal elements and their loss rate in cells pre- and post-pyrolysis, (**c**) effect of pyrolysis temperature in air atmosphere on the loss rate of metals in cells, (**d**) effect of pyrolysis atmosphere at 450 °C on the loss rate of metals in cells.

Figure 3a illustrates the pyrolysis process of PV laminates, highlighting the transition from pyrolysis to the separation of solar cells. The figure reveals that pyrolysis effectively dismantles the encapsulation structure, facilitating the separation of solar cells. This step is crucial for the subsequent analysis of valuable metals and inclusions within the solar cells. Figure 3b shows that post-pyrolysis, the levels of metal elements such as Si, Ag, Al, Ti, and Sn in the battery sheet decreased, while the levels of non-metal elements C and O increased. The calculated loss rates for Si, Al, and Ag are 5.84%, 4.32%, and 3.70%, respectively, with all mass losses remaining under 5%. This data indicates that pyrolysis has a minimal impact on the burning loss of high-value elements like Si, Ag, and Al. To further minimize metal burning loss in Si cells during pyrolysis, we analyzed the effect of pyrolysis temperature on the metal burning loss rate, as depicted in Figure 3c.

The study found that as pyrolysis temperature increased, the metal loss rates in the cells also rose. At lower temperatures, Al reacted more vigorously with oxygen than Ag, leading to greater Al loss as temperature increased. The optimal pyrolysis temperature was determined to be 450 °C. At this temperature, we investigated the impact of different pyrolysis atmospheres on metal loss rates, as shown in Figure 3d. Under vacuum conditions, the loss rates for Si, Al, and Ag were the lowest, at 4.75%, 3.62%, and 2.22%, respectively. The differences in metal loss rates across the three atmospheres were minimal. Even when pyrolysis occurred in air, the metal loss rates stayed below 6%, indicating that atmospheric oxidation loss was limited under the experimental conditions. Thus, the choice of pyrolysis atmosphere was not solely based on minimizing elemental loss. Although vacuum pyrolysis resulted in the lowest metal loss, it required additional systems for vacuum evacuation and maintenance, complicating the process. In contrast, air pyrolysis reduced air loss conditions while maintaining low metal loss. Therefore, considering the balance between metal loss, process simplification, and potential operational burden, air pyrolysis emerged as a more practical and feasible approach for loss reduction.

**Figure 4:**
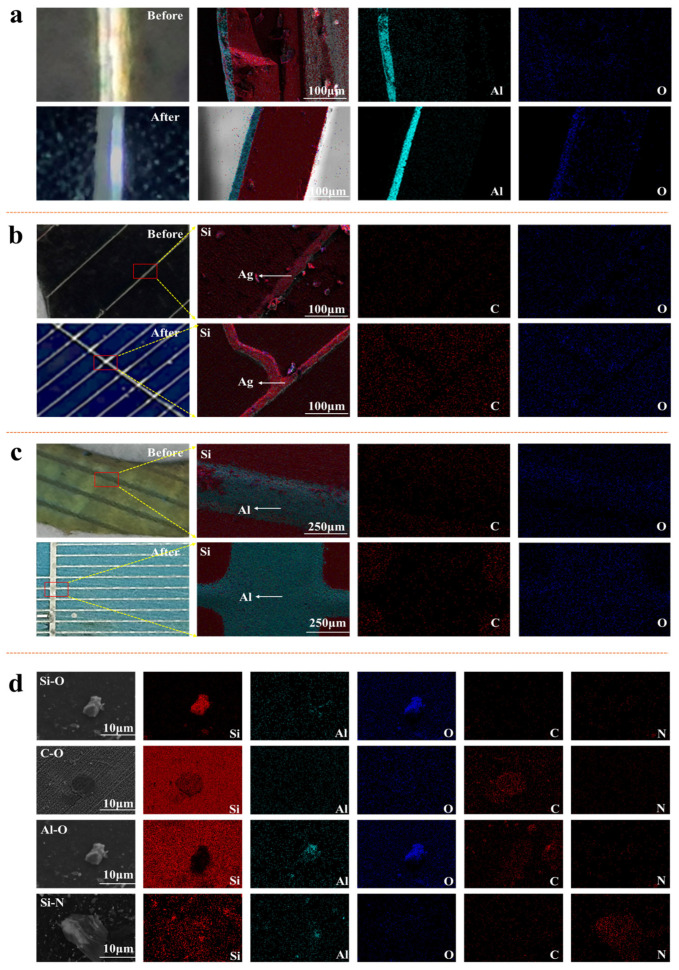
Impact of pyrolysis on C and O content in various sections of crystalline silicon solar cells: (**a**) Side, (**b**) Front, (**c**) Back, (**d**) Elemental distribution of typical inclusions in the cell after pyrolysis.

Figure 4a illustrates the presence of an aluminum oxide layer on the side cross-section of the crystalline silicon cell, which serves as the passivation interface in PERC crystalline silicon cells. After pyrolysis, the oxygen content within this aluminum oxide layer increases compared to its pre-pyrolysis level. Additionally, there is a slight increase in the overall oxygen content within the silicon wafer itself. Figure 4b shows the silver grid lines on the front side of the silicon solar cell, representing the silver front electrodes. Following pyrolysis, the overall oxygen and carbon content on this side increases. Similarly, Figure 4c reveals the aluminum grid lines on the back side, indicating the aluminum back electrodes. Post-pyrolysis, the oxygen and nitrogen content on the back side of the crystalline silicon solar cell also rises. These observations suggest that oxidation occurs in the solar cell during the pyrolysis process. Furthermore, during the air pyrolysis of the PV laminate, the thermal decomposition of the adhesive film produces trace carbides that remain on the cell surface, leading to an increase in carbon content. To investigate the impact of the pyrolysis process on the characteristics of inclusions in crystalline silicon solar cells, we first employed an electron microscope to examine typical inclusions in these cells after pyrolysis, as depicted in Figure 4d. Most inclusions, except for the relatively large Si-N type, measured under 10 μm. Si-O type inclusions typically appeared as strips or irregular particles, noted for their bright white appearance. In contrast, C-O type inclusions were primarily round and flaky, with slightly white edges and light black interiors. Al-O type inclusions generally presented as irregular particles with bright white edges and slightly darker centers. Si-N type inclusions stood out for their larger size and irregular shape, characterized by prominent surface wrinkles.

**Figure 5:**
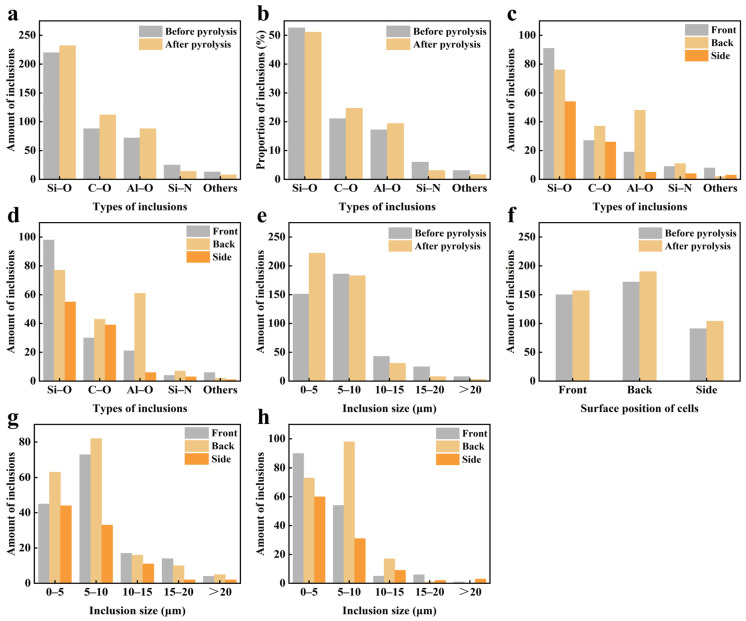
Statistics of various types of inclusions of crystalline silicon cells before and after pyrolysis: (**a**) Number, (**b**) Proportion; The proportion of different types of inclusions in different parts of the cell: (**c**) Before pyrolysis, (**d**) After pyrolysis; The number of inclusions in the cell before and after pyrolysis: (**e**) Different size ranges, (**f**) Different parts; The number of inclusions of different sizes in different parts of the cell: (**g**) Before pyrolysis, (**h**) After pyrolysis.

Nano Measurer 1.2 was utilized to statistically analyze the quantity and proportion of different types of inclusions in silicon solar cells before and after pyrolysis. Figure 5a shows that after pyrolysis, Si-O type inclusions were the most prevalent, followed by C-O, Al-O, and Si-N types, with a few other types also present. Figure 5b indicates that post-pyrolysis, the proportions of Si-O and Si-N inclusions slightly decreased, while C-O and Al-O inclusions increased. This suggests that oxygen and carbon introduced during pyrolysis facilitated the formation of these inclusions. The impact of pyrolysis on inclusion types on the cell’s front, back, and side surfaces was examined. Figure 5c,d reveal minimal differences in the quantities of various inclusion types on these surfaces before and after pyrolysis. No new inclusion types formed, and the content of existing inclusions remained largely unchanged.

We counted inclusions across various size ranges and cell sections before and after pyrolysis. Figure 5e shows a significant increase in inclusions smaller than 5 μm post-pyrolysis, attributed to the partial oxidation of silicon and aluminum, resulting in more small Si-O and Al-O inclusions. Additionally, Figure 5f illustrates an increase in inclusions on the front, back, and side surfaces post-pyrolysis. Figure 5g,h demonstrate a significant increase in small particle inclusions (size < 5 μm) on the cell’s front and side surfaces post-pyrolysis compared to pre-pyrolysis, suggesting these small inclusions formed on the surface during pyrolysis infiltrated the cell’s interior.

### 3.2. Enhancement of Inclusion Removal Efficiency in Reverse Flow Slag Washing Refining

**Figure 6:**
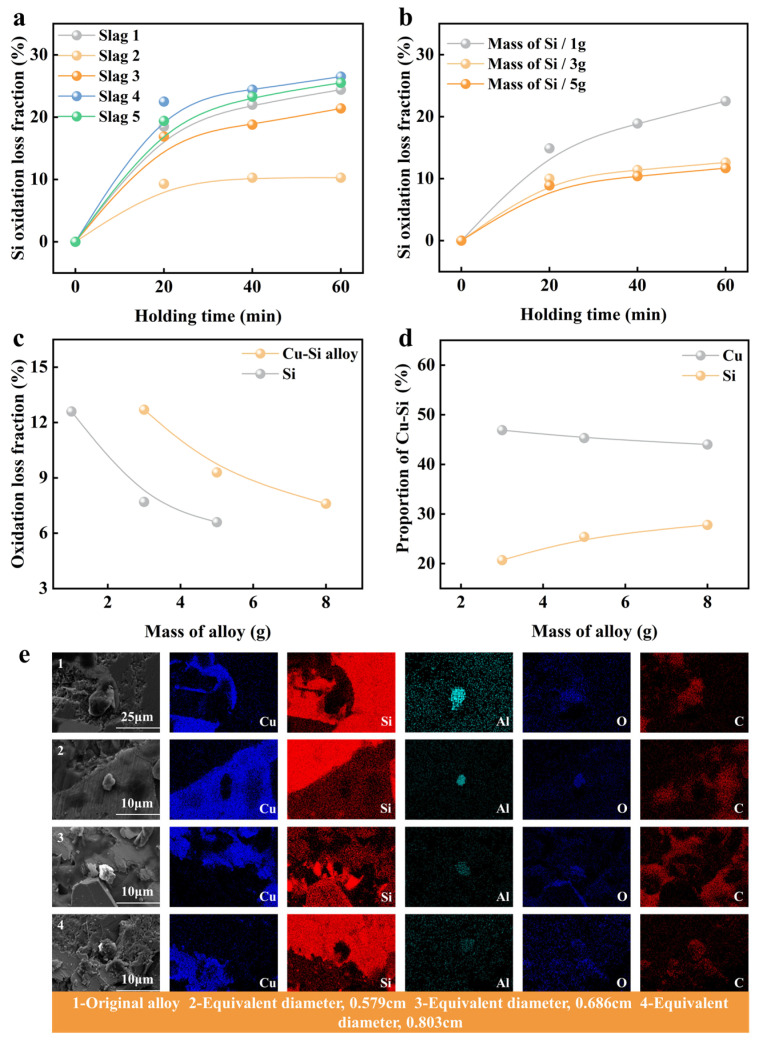
Effects of different refining conditions on the oxidation behavior and microstructure characteristics of copper-silicon alloys: (**a**) The loss of Si at different slag systems and holding times, (**b**) the loss of Si particles of different masses and holding time, (**c**) the loss of metal particles of different masses of the same slag system to Cu-Si alloy and Si, (**d**) the influence of different alloy particles of the same slag system on the composition of Cu-Si alloy, (**e**) 1-the elemental distribution of inclusions before refining, while 2, 3, 4-the elemental distribution of inclusions after refining.

The success of electroslag refining in Cu-Si alloys hinges on effectively balancing the removal of oxidation impurities with the prevention of oxidation. In our experiment, we exposed silicon particles of different masses to distinct electroslag systems for various durations before cooling them. Figure 6 presents a comparison of the slag-alloy reactions between the Cu-Si alloy and metallic silicon. Figure 6a shows the oxidation loss rates of silicon particles after refinement across five slag systems over different holding times. Notably, the NO. 2 slag system resulted in the lowest oxidation degree for the Si particles. Figure 6b details the reaction of Si particles of varying masses within the NO. 2 slag system. The highest oxidation degree occurred with 1 g particles. As the mass of Si particles decreases, their equivalent diameter reduces, increasing their specific surface area. Consequently, these smaller Si particles are more susceptible to oxidation.

Figure 6a,b demonstrate that increasing the holding time enhances the oxidation of Si particles. When the heat preservation time was 20 min, using slag NO. 2 resulted in lower burn-off during refining, regardless of whether the Si particle mass was 3 g or 5 g. Figure 6c presents the analysis of how varying masses of Si particles and Cu-Si alloys influence the oxidation loss rate of the alloy. The oxidation loss rate of metals decreased as mass increased. Notably, the Cu-Si alloy exhibited a significantly higher oxidation loss rate, indicating a more intense slag-alloy reaction. Figure 6d clearly shows that silicon loss was more pronounced during the refining of the Cu-Si alloy. However, when the alloy mass was 8 g, the silicon loss was comparatively minimal.

The elemental distributions of inclusions in the Cu-Si alloy were analyzed both before and after slag refining, with the results shown in Figure 6e. Initially, the alloy predominantly contained oval-shaped Al-O-C metal inclusions, most of which measured over 20 μm. These inclusions significantly impaired the alloy’s overall properties. After slag refining, the inclusions in the alloy were typically under 10 μm in size. As the alloy’s equivalent diameter increased, the size of these inclusions also tended to increase. Notably, the alloy with an equivalent diameter of 0.579 cm contained the smallest inclusions, which favorably supported nucleation during the refining process.

**Figure 7:**
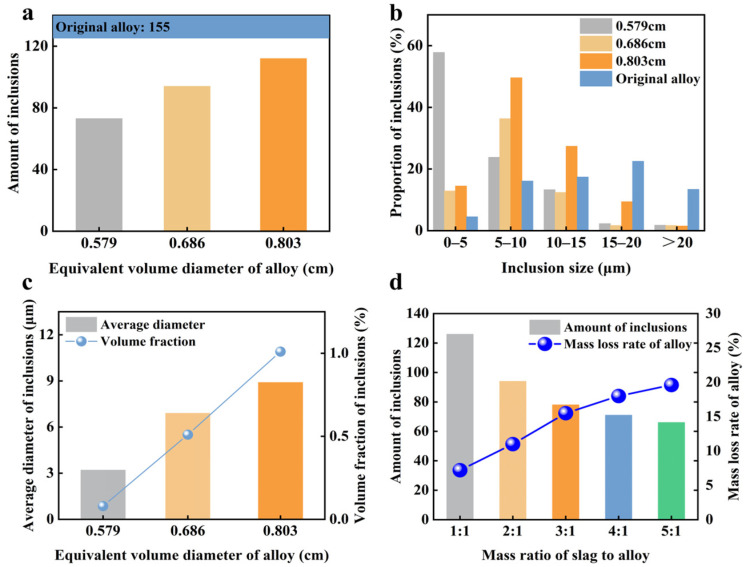
(**a**) The number of inclusions in Cu-Si alloys with different equivalent diameters before and after refining, (**b**) proportion of inclusions of different sizes before and after alloy refining, (**c**) average size and volume fraction of inclusions in Cu-Si alloys with different equivalent diameters before and after refining, (**d**) effect of the mass ratio of slag to alloy on the number of inclusions and the alloy loss rate.

Figure 7a,b illustrate the analysis of how the equivalent diameter of the alloy affects the removal efficiency of inclusions in the Cu-Si alloy. After slag refining, the number of inclusions in the alloy significantly decreases, falling from 155 before refining to a minimum of less than 80. Additionally, as the equivalent diameter of the alloy decreases, the number of inclusions gradually diminishes. This reduction occurs because a smaller equivalent diameter increases the specific surface area of the alloy droplets, thereby enhancing the reaction between the alloy and the molten slag. After slag refining, the proportion of inclusions larger than 15 μm significantly decreases, demonstrating the process’s effectiveness in removing these large-sized inclusions.

As the equivalent diameter of the alloy decreases, the peak proportion of inclusions shifts from the 5–10 μm range to the 0–5 μm range. This shift suggests a gradual reduction in the overall size of inclusions. Figure 7c presents the average diameter and volume fraction of inclusions in alloys with different equivalent diameters after slag refining. Post-refinement, the volume fraction of inclusions decreased from 6.955% in the initial alloy to as low as 0.08%, while the average inclusion size reduced from 19.5 μm to a minimum of 3.2 μm. Additionally, the data indicate that as the equivalent diameter of the Cu-Si alloy decreases, the average size of inclusions also diminishes. This implies that a smaller equivalent diameter during the slag refining process enhances the removal of large-sized inclusions. The volume fraction is expressed as follows [48]:

(3) $$V_{f}=\left[\frac{1.4\pi }{6}\right]\times \left[\frac{ND^{2}}{A}\right]$$

*D* denotes the average particle diameter, *N* signifies the number of precipitates, and *A* indicates the observed field area.

In the slag refining process, the contact area between alloy droplets and molten slag is crucial for effective inclusion removal. Increasing the slag-to-alloy mass ratio effectively enhances the amount of slag, thereby improving contact between the alloy and molten slag. This enhanced contact facilitates the floating and removal of inclusions. Figure 7d illustrates the impact of the slag-to-alloy ratio on both the number of inclusions in the Cu-Si alloy and the alloy loss rate. As the mass ratio of slag to alloy increased, the number of inclusions in the Cu-Si alloy decreased from over 120 to a minimum of 66, highlighting the significant influence of this ratio on inclusion removal.

When the slag-to-alloy ratio exceeds 2:1, the effectiveness of further increasing the ratio in removing inclusions diminishes. This is because a higher mass ratio enlarges the interfacial reaction area between the alloy and molten slag, which initially enhances inclusion removal. However, once the ratio becomes too high, the slag fully immerses the alloy, allowing a complete reaction between the slag and alloy. At this point, further increases in the ratio have minimal impact on inclusion removal. Additionally, as the slag-to-alloy ratio rises, the mass loss rate of the Cu-Si alloy also increases. The increased amount of slag intensifies the oxidation of silicon in the alloy, leading to mass loss and affecting the final yield. Therefore, to effectively remove inclusions while minimizing alloy loss, maintaining the slag-to-alloy ratio at 2:1 is optimal. At this ratio, the number of inclusions is 89, and the alloy loss rate is 11%.

**Figure 8:**
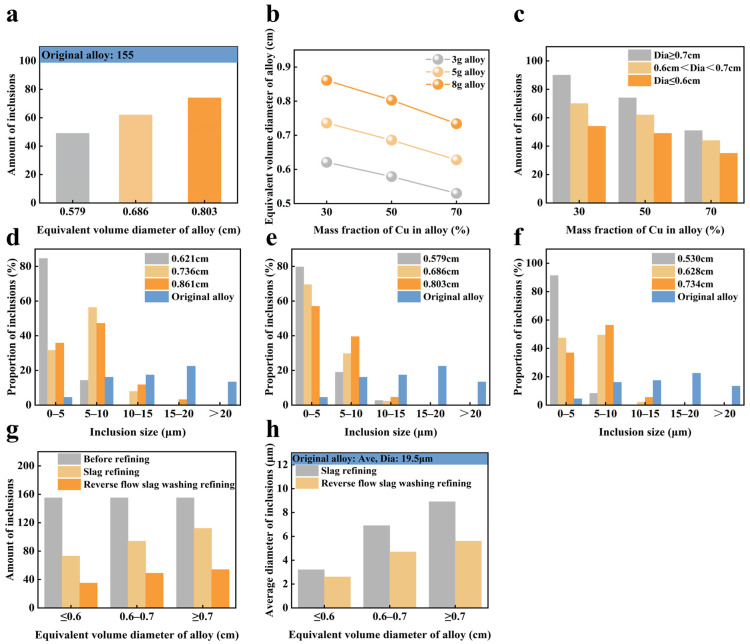
(**a**) Number of inclusions in Cu-Si alloys with different equivalent diameters before and after reverse flow slag washing refining, (**b**) calculation of equivalent diameters of Cu-Si alloys with different Cu contents, (**c**) the quantity of inclusions in refined Cu-Si alloys with different Cu contents; Effect of Cu content in Cu-Si alloy on the proportion of inclusion size distribution after reverse flow slag washing refining: (**d**) 30%Cu, (**e**) 50%Cu, (**f**) 70%Cu; Comparison of inclusion removal effects among pre- refining, slag refining, and reverse flow slag washing refining: (**g**) Amount of inclusions, (**h**) Average diameter of inclusions.

Traditional slag refining methods have limited effectiveness in removing inclusions from alloys. To improve this, we implemented a reverse flow slag washing technique. In this approach, alloy droplets traverse the slag layer, enhancing the molten slag’s capacity to desorb inclusions. The analysis of Cu-Si alloys with varying equivalent diameters after reverse flow slag washing is depicted in Figure 8a. The process reduced the number of inclusions in the alloy to approximately 50, showcasing a superior refining effect compared to traditional methods. As the equivalent diameter of the alloy decreased from 0.803 cm to 0.579 cm, the number of inclusions consistently declined. This trend suggests that reducing the equivalent diameter effectively aids in inclusion removal. Figure 8b shows that with a constant mass of a single alloy, increasing the Cu content decreases the equivalent diameter of alloy droplets during the reverse flow slag washing process. Conversely, maintaining constant Cu content while decreasing the mass of a single alloy also reduces the equivalent diameter of the droplets. Figure 8c presents the analysis of inclusion numbers in refined Cu-Si alloys across various equivalent diameter ranges. Notably, when the Cu content reached 70% or the equivalent diameter range fell below 0.6 cm, there was a significant reduction in inclusion content, with the number of inclusions dropping from a peak of 90 to a low of 35.

Figure 8d–f illustrate that as the Cu content in the alloy increases, the proportion of large-sized inclusions consistently declines. This trend facilitates the removal of large inclusions, such as C-O and Si-N types, from the Cu-Si alloy. The higher Cu content increases the alloy’s density, accelerating the descent of alloy droplets through the slag. This process enhances conditions for large inclusions to diffuse to the slag-alloy interface, promoting their reaction and migration to the slag phase. In summary, refining a Cu-Si alloy with 70% Cu content proved most efficient in removing inclusions.

This study examines the impact of pre-refining, slag refining, and reverse flow slag washing refining on the removal of inclusions from Cu-Si alloys. Figure 8g,h demonstrate that when the alloy’s equivalent diameter is ≤0.6 cm, reverse flow slag washing refining proves most effective, reducing inclusions to just 35. Initially, the average inclusion diameter was 19.5 μm. After refining, both methods decreased this diameter to below 6 μm. Notably, reverse flow slag washing refining further reduced the average inclusion diameter to a minimum of 2.6 μm when the alloy’s equivalent diameter was ≤0.6 cm.

**Figure 9:**
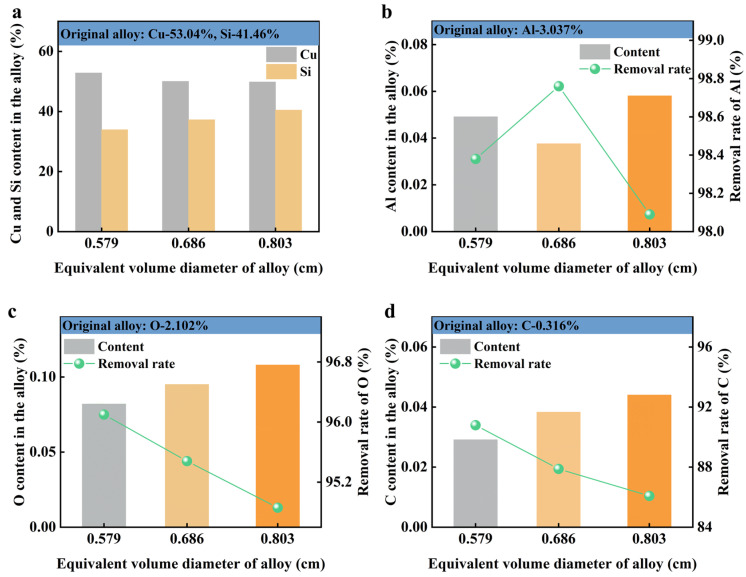
Content changes in main components and impurity elements in Cu-Si alloy before and after reverse flow slag washing refining: (**a**) Cu and Si elements, (**b**) Al element, (**c**) O element, (**d**) C element.

During the reverse flow slag washing process, numerous inclusions are removed from the alloy, significantly affecting the composition of various elements within it. To evaluate the effectiveness of impurity removal after refining, we analyzed changes in the concentrations of Cu and Si in the Cu-Si alloy, along with impurities such as Al, C, and O, before and after the refinement process. Figure 9a shows that both Cu and Si components in the alloy underwent oxidation to varying degrees. By increasing the alloy’s equivalent diameter to 0.803 cm, the loss of these components was minimized most effectively, resulting in an alloy composition of 49.83% Cu and 40.45% Si.

Figure 9b indicates that post-refinement, the Al content significantly decreased from 3.037% to below 0.06%, achieving a removal rate of over 98.09%. When the alloy’s equivalent diameter was 0.686 cm, the Al content further decreased to 0.0376%, resulting in a removal rate of 98.76%. Figure 9c,d illustrate that non-metallic impurities such as O and C were reduced from 2.102% and 0.316% to below 0.12% and 0.06%, respectively. The removal rates for O and C exceeded 94.86% and 86.08%, respectively. These findings demonstrate that reverse flow slag washing refinement effectively removes impurities like Al, O, and C from the alloy.

### 3.3. Systematic Scale-Up Experiment on NCE-ESR Refining of Cu-Si Alloy

**Figure 10:**
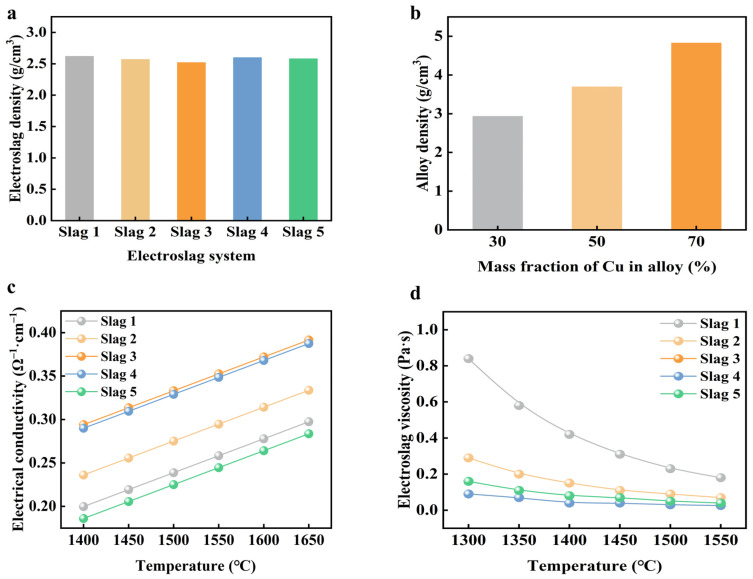
Physical parameters of five groups of slag systems: (**a**) Slag density, (**b**) Alloy density, (**c**) Slag conductivity, (**d**) Slag viscosity.

This section examines the physical properties of Cu-Si alloys, such as their high melting point, density, brittleness, and resistance to oxidation, to calculate various physical parameters of the electroslag system. These calculations are based on the slag systems designed in Section 3.2, which are intended for both slag refining and reverse flow slag system refining. The objective is to ensure the slag system meets the necessary physical performance requirements for the actual ESR refining process. In the ESR process, maintaining an appropriate density difference between the molten alloy and the liquid slag is crucial. This ensures the alloy drips through the slag pool at an optimal speed, as shown in Figure 10a,b. The density of the slag system was calculated according to Equation (1). The density variations among different slag systems were minimal, generally around 2.5 g/cm^3^. Notably, the NO. 3 electroslag exhibited the lowest density at 2.52 g/cm^3^. In contrast, the alloy’s density increased as the Cu content rose. At a Cu content of 70%, the alloy’s density reached 4.826 g/cm^3^, surpassing that of any slag system. Consequently, the alloy could effectively penetrate the slag layer.

For electroslag remelting to function effectively, the electroslag must have sufficient electrical conductivity. When an electric current passes through the electroslag, completing a circuit, it generates significant Joule heat, which quickly melts the slag pool. If the electrical conductivity is too low, the current encounters difficulty traversing the slag layer, thereby undermining the melting efficiency and stability of the slag pool. On the other hand, if the conductivity is excessively high, the slag layer becomes too thin, exposing the alloy to air and leading to increased oxidation loss. Therefore, maintaining electrical conductivity within a specific range, typically between 0.2 Ω^−1^·cm^−1^ and 0.8 Ω^−1^·cm^−1^, is crucial. Figure 10c presents the measured electrical conductivities of the five slag systems, calculated using Equation (2). It is noteworthy that as the temperature increases, the electrical conductivity of each electroslag also rises.

The melting points of the five slag systems are all below 1500 °C, while the Cu-Si alloy raw material melts at approximately 1330 °C. Therefore, the melting temperature was set at 1550 °C to ensure effective experimentation. Under these conditions, the electrical conductivity of each slag system ranged from 0.2 Ω^−1^·cm^−1^ to 0.4 Ω^−1^·cm^−1^. During the ESR refining process, the combined effects of Lorentz magnetic force and Joule force consistently agitated and refreshed the slag pool. A slag system with lower viscosity improved the fluidity of the slag pool, promoting reverse flow and facilitating slag washing. Using Factsage software, the viscosities of the five electroslag groups at various temperatures were calculated, as depicted in Figure 10d. As temperature increased, the viscosity of each electroslag system decreased, reducing resistance as the liquid alloy descended through the molten slag pool during ESR. At the refining temperature of 1550 °C, the NO. 3 and NO. 4 electroslag systems exhibited the lowest viscosities, both at 0.026 Pa·s, whereas the NO. 1 electroslag system had the highest viscosity, reaching 0.18 Pa·s.

**Figure 11:**
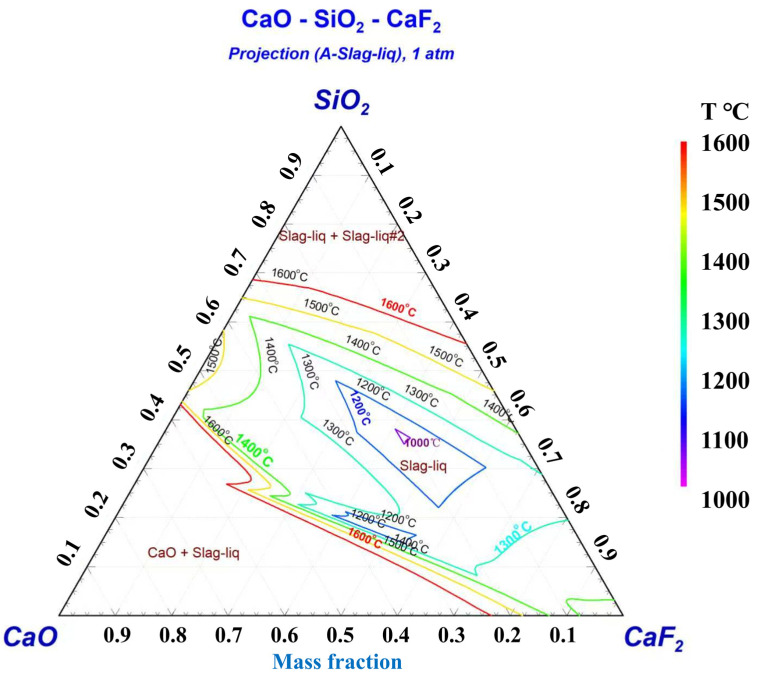
Liquid-phase diagram of the CaO-CaF_2_-SiO_2_ ternary slag system.

The liquidus isothermal phase diagram for the CaO-SiO_2_-CaF_2_ ternary electroslag system was calculated using the phase diagram module of Factsage, as depicted in Figure 11. The pale yellow area in the figure indicates the liquid phase region of the electroslag at 1500 °C. The three red dashed lines serve as auxiliary lines, representing basicity values of 0.5, 1, and 2, which correspond to the CaO to SiO_2_ ratio in the electroslag. The slag system, derived from the electroslag composition at the intersection of these red dashed lines and the pale yellow area, can melt smoothly at 1500 °C, satisfying the melting point requirements for electroslag remelting refining. Analyzing the phase diagram, the liquid phase region at 1500 °C shows that CaO content ranges from 0% to 56.78%, SiO_2_ content ranges from 21.96% to 63.56%, and CaF_2_ content ranges from 2.52% to 100%. Notably, the compositions of the five slag systems all meet the design requirements for the melting point of the electroslag system.

**Figure 12:**
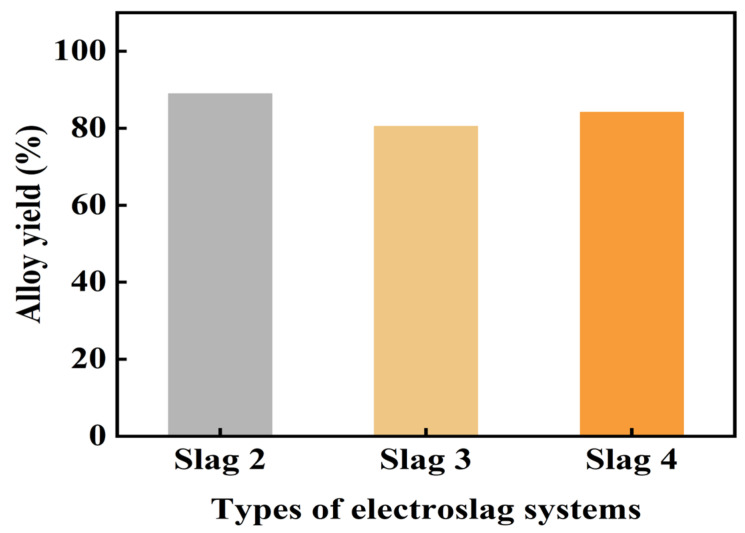
Yield rates of alloys following reverse flow slag washing refinement across various slag systems.

Electroslag NO. 2, NO. 3, and NO. 4 were selected for the reverse flow slag washing refining experiment of the optimized slag system based on their physical properties, including density, conductivity, viscosity, and melting point. Figure 12 presents the yields of Cu-Si alloys, each with identical mass, refined using these three slag systems. The findings indicated that all systems achieved alloy yields exceeding 80%. Electroslag NO. 2 notably reached the highest yield at 88.92%, whereas electroslag NO. 3 recorded the lowest at 80.45%. Therefore, electroslag NO. 2 was chosen as the optimal slag agent for the ESR scale-up experiment.

**Figure 13:**
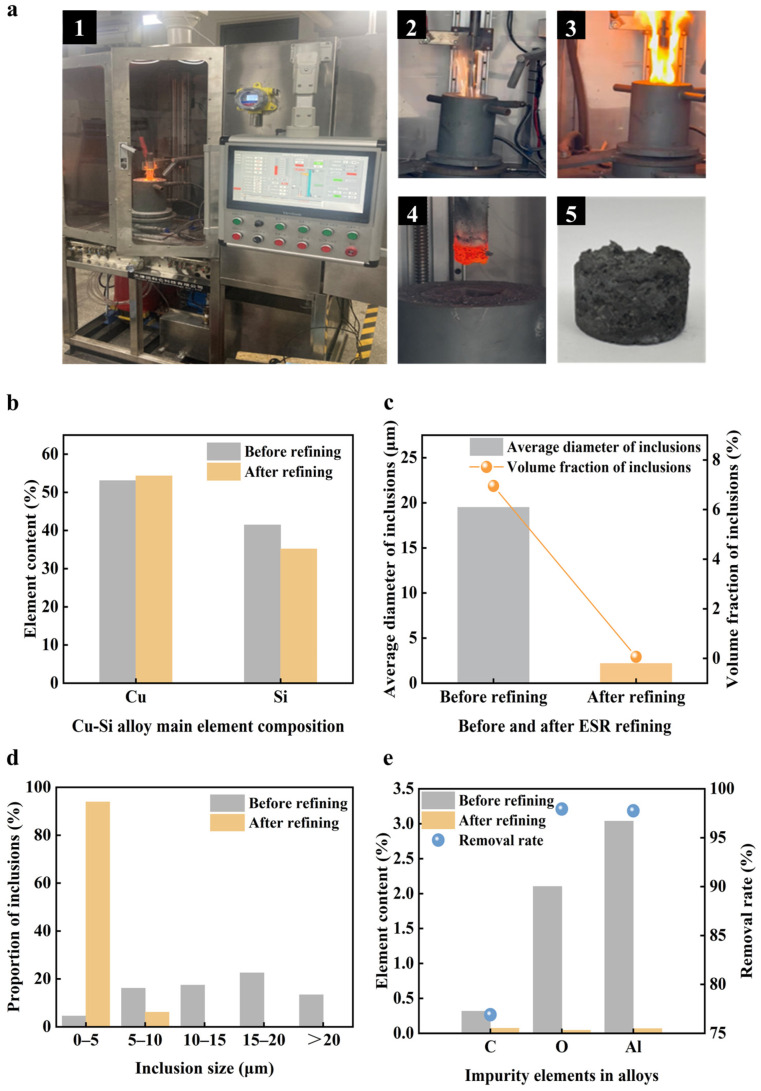
(**a**) 1-NCE-ESR furnace, 2-arcing process in the slagging stage, 3-electroslag melting process, 4-state of graphite electrodes at the end of smelting, 5-macroscopic morphology of the refined sample, (**b**) changes in the content of Cu and Si elements in the alloy before and after refining, (**c**) changes in the average diameter and volume fraction of inclusions in the alloy before and after refining, (**d**) changes in the number of inclusions in the alloy in the same plane before and after refining and the size of the distribution, (**e**) changes in the content of Al, O, and C impurities in the alloy before and after refining.

In the experiment, graphite electrodes were used to conduct current and facilitate heating and melting, as shown in Figure 13(a1). An ESR refining experiment was conducted using slag system NO. 2 and a Cu-Si alloy containing 70% Cu. For this experiment, Cu-Si alloy particles, each with an equivalent diameter of 0.579 cm, were utilized, totaling a mass of 100 g. The refining process comprised two main stages. The initial stage involved slag melting. In this phase, a graphite electrode conducted an electric current and was carefully lowered to the mold’s bottom to initiate arc starting and heating. When the electrode was approximately 1–2 cm from the mold’s bottom, an arc was generated, as illustrated in Figure 13(a2). As the electrode made further contact with the slag, the intense heat produced rapidly melted the electroslag on the surface of the cooling bottom plate, as depicted in Figure 13(a3). The subsequent phase was the remelting stage. Once a stable liquid slag pool was established, the process transitioned to remelting mode. During this stage, the height of the graphite electrode was automatically adjusted to ensure a smooth smelting process. Cu-Si alloy particles were gradually introduced into the mold. After 20 min of refining, heating was ceased, and the graphite electrode was returned to its initial position, as depicted in Figure 13(a4). The ESR sample, obtained after the alloy cooled, is illustrated in Figure 13(a5).

Figure 13b illustrates the variations in Cu and Si compositions of the alloy before and after ESR refining. The data indicate a slight increase in Cu content post-refining, while Si content decreased from 41.46% to 35.16%. This reduction in Si is attributed not only to oxidation and slag burning loss but also to the reaction between the TiO_2_ in the arc-starting agent and silicon at high temperatures, forming silicon dioxide and causing silicon loss. Consequently, the proportion of Cu increased slightly. The analysis of inclusions in alloy samples, both pre- and post-ESR refining, showed notable changes. As depicted in Figure 13c,d, the average inclusion size decreased to 2.18 μm, and the volume fraction reduced to 0.064% after refining. Remarkably, all inclusions were under 10 μm, with 93.88% within the 0–5 μm range, demonstrating the ESR’s efficacy in reducing inclusions.

Figure 13e presents the changes in impurity levels, specifically Al, O, and C, before and after refining. Post-refining, impurity levels for Al, O, and C were all below 0.08%. The removal rates were calculated as 97.75% for Al, 97.93% for O, and 76.90% for C. These results are comparable to those in silicon metallurgical refining studies, with aluminum removal efficiency exceeding 97%. For instance, Zhou et al. [54] reported a 98.1% aluminum removal rate using electromagnetic separation and slag treatment in silicon recovery from waste slag. The oxygen removal efficiency (>97%) aligns with the expected role of slag-assisted refining in enhancing melt cleanliness and removing oxide-related inclusions. In industrial silicon refining, oxygen is typically discussed alongside inclusions rather than as a standalone dissolved element. Previous studies confirm that slag refining effectively removes inclusions from silicon melt. In contrast, while the carbon removal efficiency in this study is high (>76%), it is lower than that for aluminum and oxygen, consistent with literature reports. Wen et al. [55] noted a decrease in carbon content from 5700 ppmw to 260 ppmw during oxidation refining, achieving a 95.44% removal rate. The study suggests that carbon removal is closely linked to the transformation of its states (including TiC, CaC_2_, and SiC) rather than simple oxidation. Thus, the relatively low carbon removal rate in the Cu-Si-ESR system may be due to carbon’s state and kinetic limitations during refining. Overall, the data underscore the significant impurity reduction achieved through ESR. The Cu-Si alloy electroslag system is designed to support the seamless operation of the NCE-ESR refining process, effectively minimizing the impact on primary components, Cu and Si, while efficiently removing inclusions.

## 4. Conclusions

A novel process for recycling PV modules has been introduced, utilizing NCE-ESR refining to effectively eliminate inclusions from pyrolyzed silicon cells. This method achieves highly efficient silicon purification. The key findings from our preliminary research are outlined below.

(1) Pyrolytic separation often results in the loss of metal elements such as Si, Ag, and Al in c-Si solar cells. However, performing pyrolysis in a vacuum at 450 °C significantly mitigates this loss, with burning loss rates recorded at 4.75% for Si, 3.62% for Ag, and 2.22% for Al. Interestingly, the loss rates of these metals after pyrolysis under various atmospheric conditions show minimal variation, staying within a 3% range. Even in oxygen-rich environments, the burning loss rates for Si, Al, and Ag remain below 6%. After pyrolysis, the cell predominantly contains inclusions of Si-O, C-O, Al-O, and Si-N types. The inevitable introduction of O and C during pyrolysis promotes the formation of these inclusions, increasing the proportion of C-O and Al-O type inclusions.

(2) Experiments on slag refining and reverse flow slag washing were performed on Cu-Si alloys, yielding significant findings. Under optimal slag refining conditions, the volume fraction of inclusions in the alloy decreased dramatically from 6.955% to 0.08%, and the average size of inclusions was reduced from 19.5 μm to 3.2 μm. However, this process resulted in an alloy loss rate of 11%. Notably, reverse flow slag washing was more effective than traditional slag refining in removing inclusions. During this process, the minimum number of inclusions in the Cu-Si alloy was recorded at 35, with an average diameter of less than 6 μm. It was observed that as the equivalent diameter of the alloy increased, the oxidation effect of reverse flow slag washing gradually diminished. Additionally, the impurity contents of aluminum, oxygen, and carbon were significantly reduced from 3.037%, 2.102%, and 0.316% before refining to below 0.06%, 0.12%, and below 0.06%, respectively. These reductions correspond to removal rates exceeding 98%, 94%, and 86%, respectively.

(3) The NCE-ESR equipment, developed independently, underwent a kilogram-scale amplification test to confirm the design principle’s feasibility for the alloy refining slag system. The test successfully reduced inclusion sizes to under 10 μm, with an average size of 2.18 μm, and decreased the volume fraction to 0.064%. Furthermore, the refined alloy’s impurity levels of Al, O, and C were all below 0.08%. The removal rates for Al, O, and C were 97.75%, 97.93%, and 76.90%, respectively. The proposed NCE-ESR method offers an environmentally friendly solution for the efficient and synergistic recovery of silicon resources from decommissioned photovoltaic modules.

## Figures and Tables

**Table 1:** Analytically pure reagents and content percentage (wt.%).

NO.	CaO	SiO_2_	CaF_2_
1	47.5	47.5	5
2	40	40	20
3	30	30	40
4	53.33	26.67	20
5	26.67	53.33	20

## Data Availability

The original contributions presented in this study are included in the article. Further inquiries can be directed to the corresponding authors.
